# A portable Raspberry Pi‐based camera set‐up to record behaviours of frogs and other small animals under artificial or natural shelters in remote locations

**DOI:** 10.1002/ece3.10877

**Published:** 2024-03-17

**Authors:** Jordy Groffen, Conrad J. Hoskin

**Affiliations:** ^1^ College of Science and Engineering James Cook University Townsville Queensland Australia

**Keywords:** amphibians, animal behaviour, camera, invertebrates, long‐term monitoring, non‐invasive

## Abstract

We describe a Raspberry Pi‐based camera system that is portable, robust and weatherproof, with a close‐up focus (2.5 cm). We show that this camera system can be used in remote locations with high rainfall and humidity. The camera has an Infrared LED light to film in dark places and can continuously record up to 21 days (504 h). We also describe how to make concrete artificial shelters to mount the camera in. One of the great strengths of this shelter/camera set‐up is that the animals choose to take up residence and can then be filmed for extended periods with no disturbance. Furthermore, we give examples of how shelters and cameras could be used to film a range of behaviours in not only many small cryptic amphibian species but also other small vertebrates and invertebrates globally.

## Graphical abstract

A portable Raspberry Pi‐based camera set‐up installed in artificial and natural shelters to record behaviours of terrestrial frogs and other small animals.

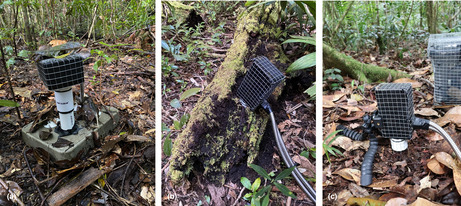

## 1 INTRODUCTION

Understanding animal behavioural responses to environmental variation and change is a frequent goal in animal ecology and has important implications for conservation (e.g. Hopkins et al., 2023; Kays et al., 2015; O'Brien et al., 2023). However, collecting behavioural data on animals, especially in remote areas, is very time‐consuming and requires a lot of effort and resources. Camera systems are increasingly used to remotely monitor wildlife because they can record behaviour over long and continuous periods and are generally non‐invasive (see reviews: Cutler & Swann, 1999; Jolles, 2021; Trolliet et al., 2014). Camera systems can be commercially built systems (e.g. Meek et al., 2014; Trolliet et al., 2014), which are ready to use and generally compact but can be expensive and have less flexibility in program settings, battery life and data storage (Cox et al., 2012; Prinz et al., 2016; Reif & Tornberg, 2006). Alternatively, they can be do‐it‐yourself assembly, which can be more flexible and cheaper (e.g. Cox et al., 2012), but can be time‐consuming to make and harder to use.

Self‐assembled camera set‐ups generally use a microcomputer, typically Raspberry Pi (www.raspberrypi.org) or Arduino (www.arduino.cc; Allan et al., 2018; Greenville & Emery, 2016; Johnston & Cox, 2017). Raspberry Pi‐based video recorders have been used in animal behavioural studies; for example, behavioural studies of the waggle dance of honeybees (Ai et al., 2017), the spacing of foraging fruit flies (Churchill et al., 2020), nematode behaviour (Nuñez et al., 2017), monitoring of mammal populations (see review Swann et al., 2004), respiration and pupil dilation in laboratory mice (Kallmyer et al., 2017; Privitera et al., 2020), avian studies assessing nest box use, parental care and other behaviours (e.g. Hereward et al., 2021; Prinz et al., 2016; Zárybnická et al., 2016). The use of Raspberry Pi units to collect video data on behaviour in amphibians has, to the best of our knowledge, only been conducted on Hellbender salamanders (*Cryptobranchus alleganiensis*) in the United States (O'Brien et al., 2023), where cameras were used in artificial aquatic shelters to described courtship and parental care behaviours and nest outcome. Self‐assembled cameras can also be combined with other sensors; for example, temperature and humidity sensors (McBride & Courter, 2019).

Artificial shelters have been proven to be a successful tool to monitor amphibian populations. They have an advantage over labour‐intensive trapping and observation methods (Sutherland et al., 2016) and reduce disturbance to the animals and their environment (Hesed, 2012). Artificial shelters built with the option of camera observation are a good way to obtain important behavioural data while minimising impacts on animals or their habitat. Once an animal is using a shelter, it can be observed for extended periods using a small camera that is serviced without any disturbance to the animal or the shelter. However, many amphibian species are small, so cameras need to focus on small subjects at close range. Furthermore, many amphibian species live in remote areas (e.g. mountain tops), so shelters and associated camera set‐ups need to be small and transportable and have long recording periods. Previous studies have made portable camera set‐ups, but the minimum focal distance is 15–25 cm (Hereward et al., 2021; O'Brien et al., 2023) and the recording time is limited to a maximum of 96 h (O'Brien et al., 2023). These cameras are of limited use for the study of small animals under shelters, and need to be serviced regularly or recordings need to be short and/or well‐spaced in time.

In this study, we describe and test a Raspberry Pi camera set‐up that is portable, robust to weather, can continuously record up to 21 days (504 h), and has a focal distance of 2.5 cm. The camera set‐up was tested on a small (average 2.4 cm long) microhylid frog species that lives among leaf‐litter, logs and rocks on remote mountaintops in north Queensland, Australia. We also describe two artificial shelters to mount the cameras in. The combination artificial shelter/camera set‐up we present could be used to collect natural history and behavioural data on any small, cryptic vertebrate or invertebrate species.

## 2 MATERIALS AND METHODS

### 2.1 Camera set‐up

**FIGURE 1:**
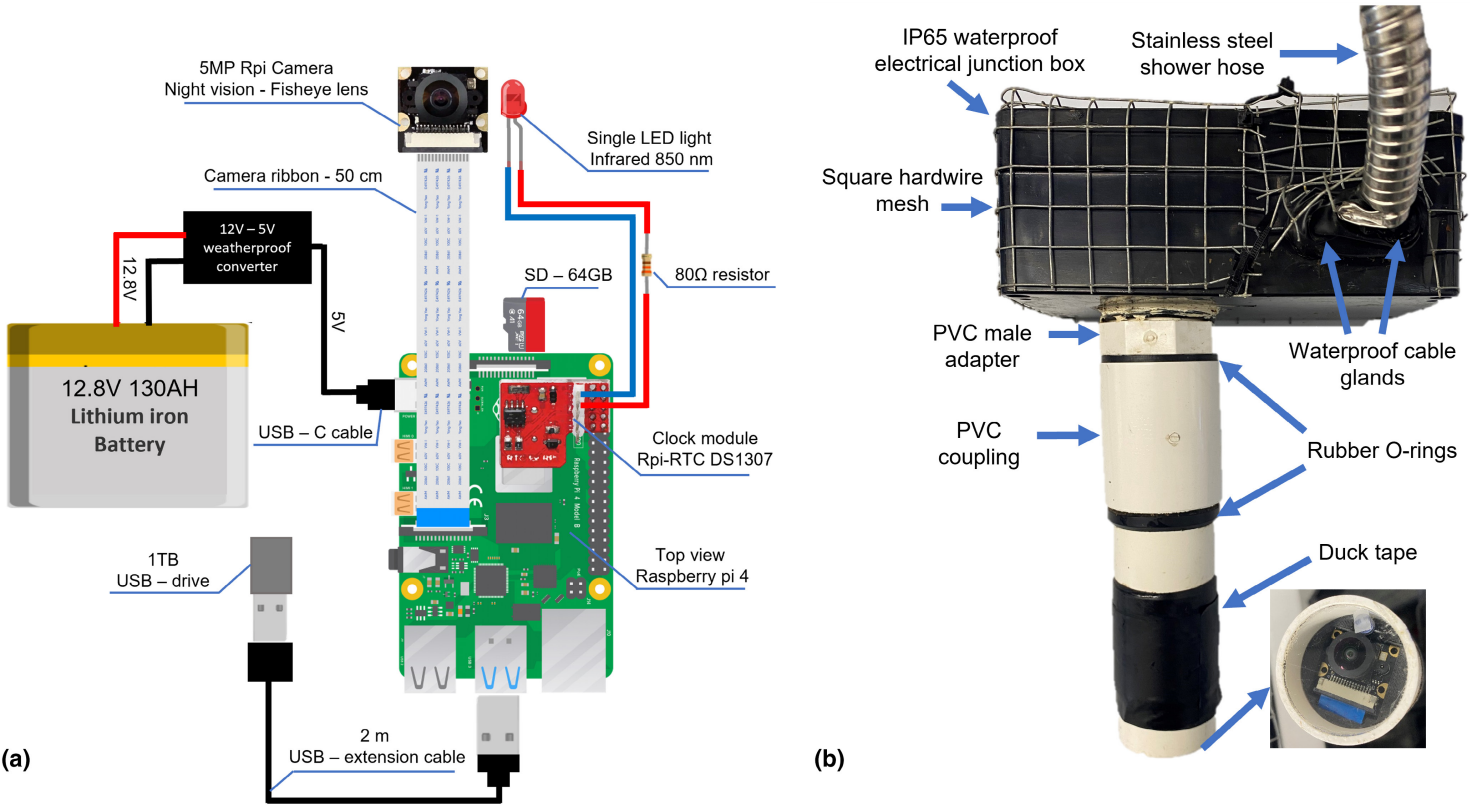
Circuit diagram of the camera set‐up (a), and a photo of the camera housing (the Raspberry Pi unit is inside the black electrical junction box (b)).

For the camera set‐up, we used a Raspberry Pi 4 microcomputer circuit board with a 5‐megapixel 1080P camera (25 × 24 × 17 mm) with a 130° adjustable night vision fisheye lens (focus 2.5 cm) and a single 5 mm infrared LED light (Figure 1). We removed the round part of the infrared LED light using a belt sander and added a small semi‐transparent piece of sticky tape to diffuse the light throughout the filming chamber (Figure 1b). A Rpi‐RTC DS1307 clock module keeps accurate time while not connected to the internet. The microcomputer was placed in an IP65 waterproof electrical junction box (158 × 90 × 60 mm) with four 1 g silica gel desiccant packets. Three holes were drilled in the junction box: two on the long side for waterproof cable glands (fits cables between 3 and 6.5 mm) and one on the bottom for the PVC male adaptor (SKU 436‐010; 3 cm). The power source cable (USB type C) and the USB extension cable were inserted individually through one of the two cable glands and together into a 1.5‐m long stainless‐steel shower hose to prevent damage by rodents (which chew exposed rubber or plastics). On the side of the stainless‐steel hose, the cables were inserted individually through cable glands in an IP67‐rated waterproof PVC box (38.8 × 28.9 × 24.3 cm). In this box, the USB power cable is connected to a power converter (12 to 5 V) connected to a 130 Ah LiFePO4 lithium battery (10 kg). A 1 TB USB storage device is connected to the USB extension cable (Figure 1a).

Trials by Mouy et al. (2020) have found that USB storage used more energy than SD card storage, therefore lowering battery life, and USB storage was less reliable because of its more fragile connection. However, O'Brien et al. (2023 ) and Kallmyer et al. (2017) successfully used 64 and 32 GB USB storage device in their study, respectively. In this study, we chose a 1 TB USB storage device because the USB storage device and battery are separated from the microcomputer; which has the benefit that the USB storage device and battery are replaced in the field after 21 days (often during rain and 90% >humidity), we do not have to open the box with the microcomputer and camera preventing potential damage by moisture. The total cost for the 21‐day camera set‐up is AUS$790.75 (Table 1).

**TABLE 1:** Items, and their costs, required to build a camera set‐up that can record continuously for up to 21 days.

Camera set‐up items	AUS$	US$	Items to waterproof the set‐up	AUS$	US$
5‐megapixel 1080P camera	6.90	4.62	Plastic waterproof (IP67) electrical box	14.56	9.74
Camera ribbon (30 cm or 50 cm)	2.03	1.36	Waterproof (IP65) container 18 L	15.68	10.49
Raspberry Pi model 4	75.84	50.75	Valve adapter 32 mm	5.75	3.85
64 GB microSD card	21.00	14.06	PVC coupling 32 mm	3.63	2.43
1 TB USB drive	160.00	104.56	Rubber O‐rings	1.04	0.70
12 V 130 Ah LiFePO4 lithium battery	402.99	263.35	PVC pipe 32 mm	2.50	1.67
Coin cell battery	1.08	0.72	M12 cable gland waterproof connector	2.60	1.74
Rpi‐RTC DS1307 clock module	3.75	2.51	Square wire mesh 1 cm x 1 cm	1.87	1.25
Weatherproof 12 V‐5 V converter	23.99	16.06	Metal shower hose	11.00	7.36
USB‐C cable (2 m)	14.98	10.03	Total	58.63	39.23
USB extension cable	16.98	11.36			
LED–Infrared 850 nm	1.70	1.14			
Jumper wires 20 cm	0.88	0.59			
Total	732.12	481.11	Combined total	$790.75	$520.34

**FIGURE 2:**
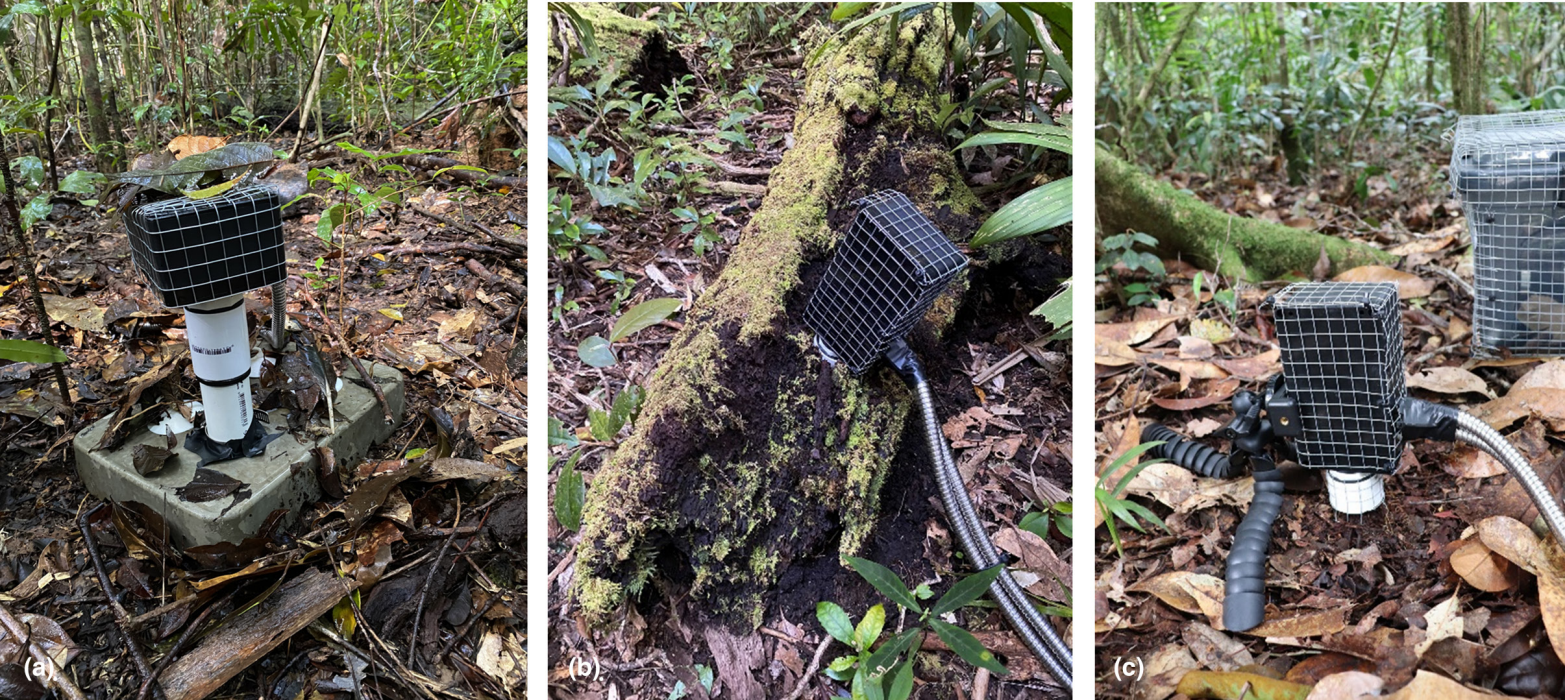
Example of how the camera is placed in an artificial concrete shelter (a), a natural log (b) and in leaf litter (c).

**FIGURE 3:**
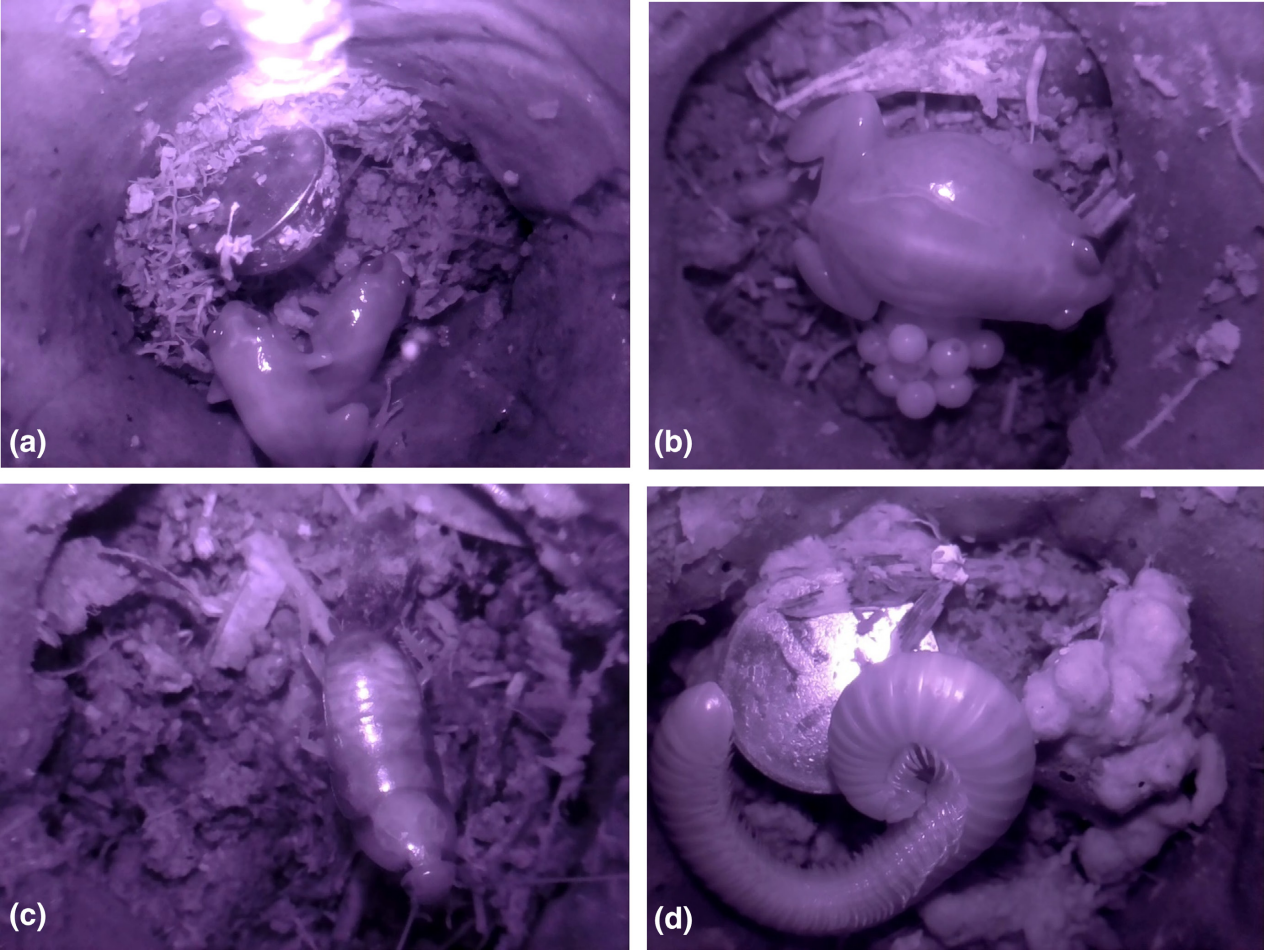
Video screenshots of animals in chambers of the concrete shelters. (a) Two *Austrochaperina robusta* next to the temperature and humidity data logger (Hygrochron Ibutton). (b) A male *A. robusta* sitting on his eggs (parental care). (c) A cockroach and (d) a millipede grooming itself.

The camera length can be easily adjusted for different habitats. For example, when filming animals under leaves or artificial shelters (Figure 2), we used the camera set‐up with the camera directly placed in the PVC male adaptor (4 cm width) and the lens focusing at 2.5 cm. To ensure that water could not enter the PVC male adaptor, a PVC coupling was placed with a glass lens glued inside and a rubber O‐ring was placed between the connections. This way, if necessary, we could still change the focus of the camera by removing the coupling. For filming under natural or artificial wood shelters (‘logs’; see paragraph ‘artificial shelters’ below), we use a 20‐cm long and 4.2‐cm wide PVC pipe in the converter, which goes over the male adaptor. The camera and the infrared LED light were held in place at the end of the PVC pipe with a 3D‐printed holder against the glass lens. Both the camera and battery box were covered with square mesh (mesh size 1 × 1 cm) to protect the plastics from chewing by rodents. To record the temperature and humidity a datalogger (Hygrochron iButton) was placed in the chamber when the camera was recording (Figure 3a).

The Raspberry Pi was coded to start recording when it powered on, and for continuous video recordings of 15 min each. These recordings run back‐to‐back but a 15‐min duration was chosen to keep the file size of each recording manageable (O'Brien et al., 2023). The ‘continuous’ recording was programmed to stop after 21 days (504 h). Videos were saved to a 1 TB USB storage device, creating folders of the month followed by folders of the day. The videos themselves were labelled with time (hour.minute.second; Appendix 1; for detailed instructions on how to program the Raspberry Pi see e.g. Hereward et al., 2021; Youngblood, 2019).

### 2.2 Artificial shelters

We designed the artificial shelters based on the preferred nesting/sheltering microhabitat of microhylid frogs, as described by Felton et al. (2006) for *Cophixalus ornatus* (now *C. australis*; Hoskin, 2012). Sheltering and nesting microhabitats are essentially small crevices or holes in soil or wood, or under leaf litter, logs or rocks. The objective was to make artificial shelters that contain small spaces and can have a camera inserted to film any of these spaces (i.e. a ‘chamber’) that is in use.

**FIGURE 4:**
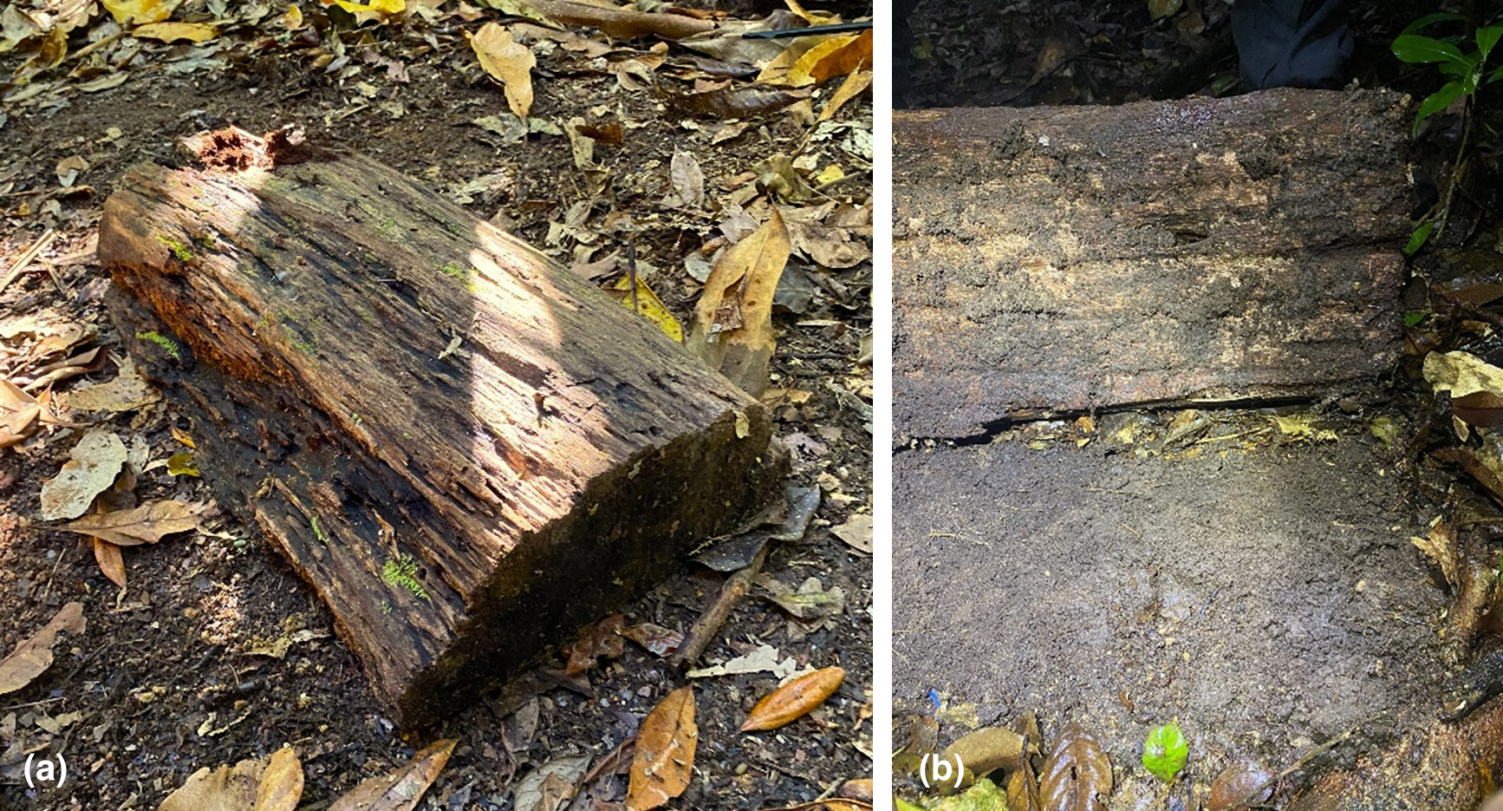
Wooden ‘natural’ shelter in place (a), and photo showing the underside of the natural shelter installed in the field, the ground is cleared from leaves before the shelter is placed (b).

Two artificial shelter types were constructed, one using wood and one using concrete. The wooden shelters were made by splitting logs found at the site (e.g. from a tree fall near a road or path; Figure 4a). By splitting the log in half, we produce a flat surface to place against the ground (Figure 4b). The resulting log size (i.e. surface in contact with the ground) was an average of 60.4 cm in length × 18.5 cm in width. When a frog occupied the shelter and we decided to film, a 42‐mm wide hole was drilled through the log to fit the camera. The diameter of the drill hole reflected the size of the end of the camera so that it can be neatly inserted. We also sourced sink plugs (40 mm diameter), which sit atop a 40 mm wide and 35 mm long piece of PVC pipe. These plugs were used to block the hole when the camera was ultimately removed. The plug has concrete packed into it to better buffer the temperature in the nest chamber.

**FIGURE 5:**
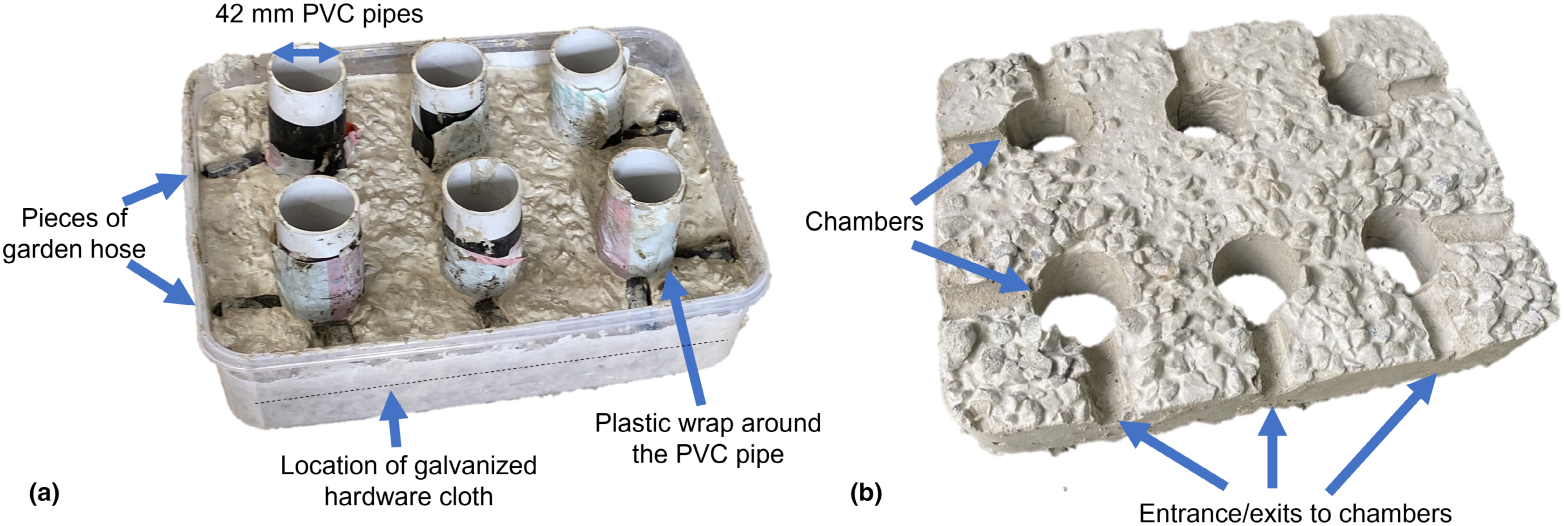
Construction of an artificial concrete shelter (a), and photo showing the underside of the resulting concrete shelter, with the six chambers and associated entrances/exits (b).

The concrete artificial shelters are rectangular concrete blocks (29.5 × 22.5 × 4.5 cm), with six rounded (42 mm diameter) nesting chambers, each with two entrances/exits (Figure 5b). A plug hole is present above each nesting chamber so that the plug (same as in wooden shelter) can be lifted to inspect the nesting chamber. We constructed concrete artificial shelters using a four‐two‐one concrete mixture (four parts crushed rock; two parts sand and one part cement). One‐third of the concrete was poured in a 4‐L Tupperware container, and then six 42‐mm wide plastic‐wrapped PVC pipes were equally distributed in the concrete, placed vertically to make chambers and a galvanised 1 × 1 cm mesh hardware cloth was placed around them. The other two‐thirds of the concrete was then poured into the Tupperware container around the PVC pipes. Pieces of garden hoses (each 3 cm long) were placed in the concrete where the entrances/exits going to be (Figure 5a). The whole Tupperware container was then placed on a vibrating plate to remove air bubbles. After drying for at least 48 h, the PVC pipes and garden hose pieces were taken out of the concrete. The shelters were then soaked in water for 3 days and then dried completely in the sun before being deployed in the field. The cost of making the shelters was AU $20, per mould (4 L Tupperware, 42  × 100 mm PVC pipe, and 10 pieces of 3 cm garden hose), AU$3 for the concrete shelter (concrete, sand, crushed rock and galvanised mesh hardware cloth) and about AU$18 for the six lids per shelter (sink plugs, PVC pipe, screws and concrete mixture; Table 1). So, once you have the moulds, which are reused, the total cost per shelter is around AU$21 (US$14), with potential labour costs not included (Table 1).

### 2.3 Testing the cameras and artificial shelters

**FIGURE 6:**
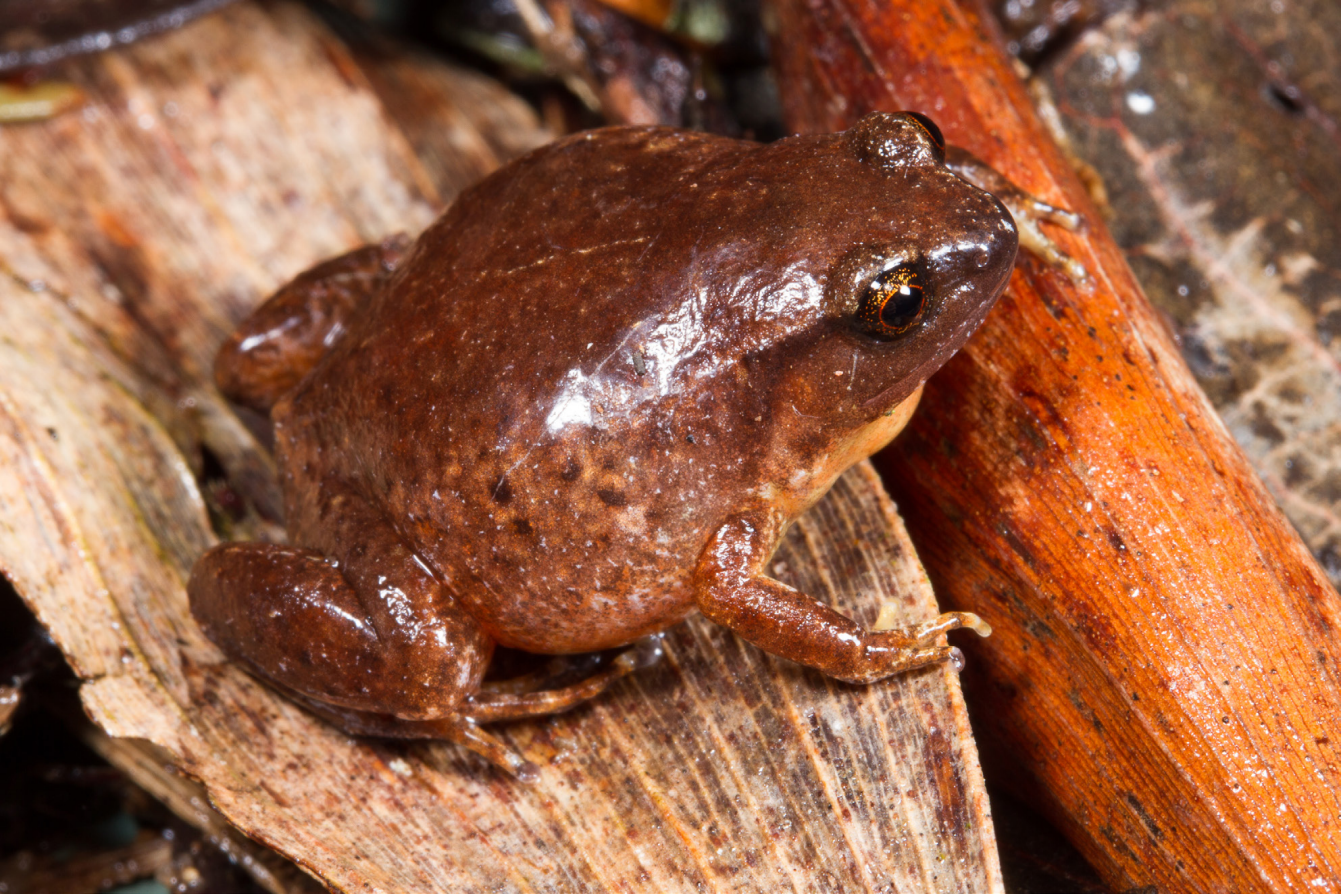
A male robust whistling Frog, *Austrochaperina robusta* (Photo by Stephen Zozaya).

The camera set‐up and artificial shelters were tested in the Paluma Range (north‐east Queensland, Australia), where a small (average 2.4 cm body length) microhylid frog (Robust Whistling Frog *Austrochaperina robusta*; Figure 6) occurs at high abundance. This species is restricted to mid‐elevation and upland rainforest, living among the leaf‐litter and under logs and rocks (Hoskin & Hero, 2008). It is extremely cryptic, other than the loud whistling calls of males calling in or after rain. *Austrochaperina robusta* is a terrestrial breeding frog, with direct development (embryos develop to metamorphosis within the jelly capsule), and the small clutch is laid in leaf‐litter and under logs and rocks and is attended by an adult frog (Hoskin, 2004). Details of breeding biology, including the role of the adult in caring for the eggs and metamorphs, as well as their year around microhabitat use are unknown due to the small size and cryptic lifestyle.

Thirty artificial concrete and 30 wooden shelters were placed in a known high‐density *A. robusta* area between November 2022 and January 2023. To test the most risk‐prone parts, switching out batteries and USB storage device in the field, we used 30 mAh 12 V batteries that can run up to 5 days. Cameras were deployed 16 times during the subsequent breeding season (21 November–23 February), with a minimum deployment of a camera being 3 days and a maximum deployment being 30 days at a single shelter. During this period, the batteries and USB storage device of the cameras were changed every 3–5 days. This involved a total of 62 battery and USB changes. The camera system was removed when the frog was not seen for 2 days or more.

## 3 RESULTS

Twenty‐four of the 30 concrete shelters and 19 of the 30 wooden shelters were occupied at least once by *A. robusta* during the field test period (80% and 63%, respectively). Cameras were placed in 11 shelters, each of which had a different *A. robusta* individual (based on shelters being occupied and the distance between the shelters). The film quality was generally excellent, including good lighting (Figure 3), and a total of 1378 h of video footage with an *A. robusta* in view was generated. Ten individuals stayed longer than 5 days in a chamber that was being filmed, while three stayed over 30 days (multiple battery changes). This suggests that the camera was accepted by the frogs, and not a source of disturbance. In addition to frogs, various other species sought refuge under the artificial shelters and were captured in the video footage. These included other small vertebrates (skink species) and invertebrates such as ants, earthworms, velvet worms, cockroaches and millipedes.

Although the cameras were thoroughly tested before being deployed in the field, a few issues arose during field testing. In two camera set‐ups, the dates and times displayed on the video files were inaccurate after batteries were replaced. To resolve this issue, we reran the codes for the clock, which successfully fixed the problem for one camera. However, we had to replace the clock module in the other camera. In one camera set‐up, the brightness of the Infrared LED light decreased in some videos before eventually turning off. This occurred towards the end of the expected video duration and was likely due to the lower voltage available. The issue was resolved by changing the battery and always using fully charged batteries. The total recording duration varied based on the level of recorded animal activity, with higher activity resulting in larger file sizes and more processing power, leading to a quicker depletion of USB storage space and battery life. The final issue was that despite adjusting and testing each camera's focus before deployment, some cameras produced out‐of‐focus video. This was primarily attributed to the camera sliding down too deep (and hence out‐of‐focus) in the shelter set‐up. This was sometimes due to the camera hole in the artificial shelters being slightly wider than the camera PVC pipe, causing the camera to slide down too far. In other cases, it appeared that the camera had been moved during filming, probably due to disturbance from wildlife such as brush turkeys or feral pigs. To address these issues, we applied multiple layers of Duct tape around the camera PVC pipe to secure it in place and prevent any sliding or moving. Another common cause of unfocused videos was the gradual elevation of the soil beneath the camera due to the burrowing behaviour of earthworms, but this could not be prevented.

We initially envisaged that the camera set‐up could be damaged by moisture. They were sitting in a wet rainforest environment with regular heavy rain, and the 62 battery and USB storage device changes were all performed in >90% humidity and sometimes during rain. However, no water damage was observed on the cameras, batteries, or USB storage device. This was even the case for one of the cameras which spent time with its lens end underwater when one of the chambers was flooded during heavy rain. The camera kept recording and was not damaged.

## 4 DISCUSSION

In this paper, we have described and demonstrated the successful building and testing of a Raspberry Pi‐based camera that produces high quality close‐up (2.5 cm) videos and is portable and weatherproof. The camera set‐up can record continuously for up to 21 days (on a single battery and USB storage device) in remote locations, and we have demonstrated how they can be used in natural settings or paired with artificial shelters. The cameras could be used for many different research topics, in many different species and settings. Collecting long‐term continuous behavioural data, especially in the field and from small species, is very intensive and often logistically not possible (e.g. at night or when the animal is in its shelter). This camera set‐up will enable behavioural data to be collected on the natural history of small and cryptic species.

A benefit of the camera is that you have control over programming when to record. In this trial, we programmed the camera to continuously record for up to 21 days. We recommend starting with this method to get an idea of the species daily time budget. However, the camera can be coded to record whatever data is required for the specific research questions; for example, for 30 min every 4 h, or only day‐ or night‐time. Specific recording periods will increase battery life and increase the camera data collection time. The self‐assembled camera set‐up could be combined with sensors such as temperature and humidity loggers (McBride & Courter, 2019). We deployed the cameras on a mountain in the tropical rainforest which has high rainfall (average annual rainfall of 2534.7 mm) and humidity during the summer months of >90% 24 h a day. We therefore did no attach temperature and humidity sensors because adding these would have required extra holes to be drilled, increasing the chance of moisture entering the set‐up. Adding sensors would also decrease the battery life. The goal of this study was to build a camera set‐up that is robust, extremely weatherproof, with as long as possible recording time and relatively cheap to make. We did use separate temperature and humidity sensors (Hygrochron iButtons), which are robust and do not connect to the camera set‐up or battery (Figure 3a).

We strongly recommend testing the recording time for each target species and environment before deploying in the field. Recording durations will vary due to differences in ambient temperatures and the amount of animal activity recorded, potentially resulting in shorter‐than‐anticipated recording duration. For instance, when recording individuals of *A. robusta* without a nest, the camera set‐up captured footage for up to 21 days (504 h). However, when recording an *A. robusta* individual with an egg clutch, the set‐up stopped recording after 15 days (358 h). This difference can be attributed to differences in behaviour – individuals without a clutch showed limited movement during the day and often left the chamber and were out of camera view at night (between 18:00 and 6:00 h), compared to the individual with an egg clutch that remained in the chamber and displayed more activity (parental care). The latter resulted in larger file sizes and increased processing power, and a more rapid depletion of USB storage space and battery life. While not tested in this study, it is known that ambient temperatures can impact battery performance. Extreme temperatures, hot or cold, may affect the efficiency of the battery. Furthermore, the quality and age of the battery can influence its overall performance, older or low‐quality batteries may not hold a charge as well. Recording times can be increased using lower resolutions and frame rates, which require less processing power and storage space. This trade‐off between video quality and maximum recording time will need to be assessed depending on the goals of the project.

The artificial shelters proved to be very successful because they were voluntarily occupied by the frogs in high numbers and enabled filming at optimal distance. Importantly, they also enabled extended behavioural observations without influencing the frog's behaviour. Obtaining sheltering and breeding information in a species like this would otherwise require regular turning of logs and other cover, disturbing (and potentially injuring) the frogs and damaging the micro‐habitat. Due to this success, we now have increased the number of shelters and expanded the research to other upland areas, with the objective of recording the breeding behaviours and breeding success of Critically Endangered microhylid frog species. While both artificial shelter types (concrete and log) were successful, filming and maintenance of the concrete shelters was easier, due to the sturdier and stable material, and the predrilled holes. In addition, the log artificial shelters will disintegrate and fall apart with time and will need to be replaced. In contrast, the concrete shelters will last and will increasingly ‘integrate’ with the environment (e.g. moss growing on them, leaf‐litter falling on them).

During our trial many other species of small animals occupied the chambers and were filmed. Of particular interest were cockroaches (*Periplaneta australasiae*) that occupied the shelters for short periods of time. We discovered that the infrared LED light used in the camera set‐up illuminate through the exoskeleton of the cockroaches, showing the internal organs (Figure 3c). In one set of filming, the infrared LED light showed that one of the cockroaches had a gut parasite that could clearly be seen moving internally. This shelter/camera set‐up could be used to study, for example, the prevalence of gut parasites in cockroaches in different habitats, the development of the gut parasite and the effect on the cockroach.

A strength of this shelter/camera set‐up is that the animals choose to take up residence and were then filmed with almost no disturbance. Servicing of the battery and USB storage device does not disturb the camera end or shelter. We suggest these shelters and cameras could be used to film a range of behaviours (including, parental care, shelter use, species interactions and courtship behaviour) in many cryptic amphibian species and other small vertebrates and invertebrates, globally.

## AUTHOR CONTRIBUTIONS

**Jordy Groffen:** Conceptualization (lead); funding acquisition (equal); investigation (lead); methodology (lead); writing – original draft (lead); writing – review and editing (lead). **Conrad J. Hoskin:** Funding acquisition (equal); resources (equal); supervision (lead); writing – review and editing (equal).

## FUNDING INFORMATION

This work was funded by the Holsworth Wildlife Research Endowment Grant (Ecological Society of Australia Ltd), the Skyrail Rainforest Research Fund (Skyrail Rainforest Foundation), and a Ric Nattrass Research Grant (Queensland Frog Society Inc.).

## Data Availability

All data and code are available in the main text or the supplementary material: Table 1 and Appendix 1.

## APPENDIX 1 Codes

### 1.1

*#This code imports the programs and hardware necessary for the code that follows*:

import os
import picamera
from datetime import datetime
import time

*#LED light*
import RPi.GPIO as GPIO
import time
GPIO.setmode(GPIO.BCM)
GPIO.setwarnings(False)
GPIO.setup(17, GPIO.OUT)
print ("LED on")
GPIO.output(17,GPIO.HIGH)

*#video run time‐> 900 sec (15 minutes) videos for 21 days*
seconds_per_video = 900
ALLOWED_RUN_TIME =  (60*60*24*21)# sec

*#Change “NAMEUSB” to the name of the USB you use*
def main():
rootdir = "/media/pi/NAMEUSB/"

*# Wait for the USB drive to be mounted to prevent errors*
while not os.path.ismount(rootdir):
time. sleep(1)
*# Creating folders by date to store the files*
start_date = datetime.now()

monthdir = rootdir + str(start_date.month) + "/"
daydir = monthdir + str(start_date.day) + "/"

print("It is currently: {}".format(start_date.strftime("%d_%m_%y_%H%M%S")))

check_create_directories(rootdir, monthdir, daydir)

camera = picamera.PiCamera()
camera. framerate=15
camera.exposure_mode=';night'
camera. resolution=(1640,1232)
run = True
while(run):

timestamp=datetime.now()

run = True if ((timestamp ‐ start_date).seconds <= ALLOWED_RUN_TIME) else False

rootdir, monthdir, daydir = check_dates_and_directories(start_date, timestamp, rootdir, monthdir, daydir)

filename = timestamp.strftime("%H_%M_%S")

try:
camera.start_recording(daydir+f"{filename}.h264")
print(f"starting 15sec recording called: {filename}.h264")
camera.wait_recording(seconds_per_video)
camera.stop_recording()
except KeyboardInterrupt:
print("User asked to stop. Stopping!")
camera.stop_recording()
exit()

def check_dates_and_directories(old_date, new_date, rootdir, monthdir, daydir):
if old_date == new_date:
return rootdir, monthdir, daydir
print("Date matches!")
else:
print("Creating new folders as needed!")
rootdir = "/media/YOURUSB/"
monthdir = rootdir + str(new_date.month) + "/"
daydir = monthdir + str(new_date.day) + "/"
check_create_directories(rootdir, monthdir, daydir)
return rootdir, monthdir, daydir

def check_create_directories(rootdir, monthdir, daydir):
if(os.path.isdir(rootdir)):
print("root directory exists, changing to it")
os.system("cd " + rootdir)
else:
print("creating root directory and changing to it")
res = os.system("mkdir " + rootdir)
print(res)
os.system("cd " + monthdir)

if(os.path.isdir(monthdir)):
print("month directory already exists, changing to it")
os.system("cd " + monthdir)
else:
print("creating month directory and changing to it")
res = os.system("mkdir " + monthdir)
print(res)
os.system("cd " + monthdir)

if(os.path.isdir(daydir)):
print("Day directory already exists, changing to it")
os.system("cd " + daydir)
else:
print("creating day directory and changing to it")
res = os.system("mkdir " + daydir)
print(res)
os.system("cd " + daydir)

if __name__ == "__main__":
main()
